# Evaluation of the effect of different sedative doses of dexmedetomidine on the intestinal motility in clinically healthy donkeys (*Equus asinus*)

**DOI:** 10.1186/s12917-022-03376-4

**Published:** 2022-07-14

**Authors:** Marwa Abass, Hussam Ibrahim, Hakan Salci, Mohamed A Hamed

**Affiliations:** 1 Department of Surgery, Anesthesiology, and Radiology, Faculty of Veterinary Medicine, Mansoura University, Mansoura, 35516 Egypt; 2 Department of Internal Medicine, Infectious and Fish Diseases, Faculty of Veterinary Medicine, Mansoura University, Mansoura, 35516 Egypt; 3 Department of Surgery, Faculty of Veterinary Medicine, Uludag Universitesi, Bursa, Turkey; 4 Department of Surgery, Anesthesiology and Radiology, Faculty of Veterinary Medicine, Aswan University, Aswan, Egypt

**Keywords:** Motility, Dexmedetomidine, Gastrointestinal tracts, Ultrasonography, Donkey

## Abstract

**Aim:**

Gastrointestinal effects of different doses of dexmedetomidine in donkeys are still unidentified. The current study aimed to evaluate the impact of different doses of dexmedetomidine on the motility of selected parts of the gastrointestinal tracts in donkeys using transabdominal ultrasonography.

**Materials and methods:**

An experimental crossover study was conducted on 30 healthy donkeys of both sexes (15 males and 15 females; 160 ± 60 kg). With a two-week washout period, each donkey received an injection of either a normal saline solution or three different doses of dexmedetomidine (3, 5, and 7 μg/kg, respectively). All medications were administered intravenously in equal volumes. The contractility of selected intestinal segments (duodenum, jejunum, left colon, right colon, and cecum) was measured 3 min before administration (zero time) and at 15, 30, 45, 60, 90, and 120 minutes after administration.

**Results:**

Small and large intestinal motility was within the normal ranges before IV injection of normal isotonic saline or dexmedetomidine at a dose of 3, 5, and 7 μg/kg.

Two Way Repeated Measures ANOVA output of the data displayed a statistically significant the between time and treatments for the contractility of each of the duodenum (*P* = 0.0029), jejunum (*P* = 0.0033), left colon (*P* = 0.0073), right colon (*P* = 0.0035), and cecum (*P* = 0.0026), implying that the impact of treatment on the gastric motility varied among different time points. The simple main effect analysis revealed that the IV dexmedetomidine at 3, 5, and 7 μg/kg doses significantly inhibited (*P* ≤ 0.01) the bowel contractility compared to the administration of isotonic saline.

**Conclusion:**

Dose-dependent inhibitory effect of dexmedetomidine on intestinal motility was reported in donkeys following intravenous administration. This inhibitory effect on intestinal motility should be considered in clinical practice*.*

## Background

Many surgical procedures on horses are performed using standing sedation and local blocks to avoid the risks of general anesthesia [1, 2]. In equines, alpha-2 adrenergic receptor agonists are commonly utilized for sedation, analgesia, and muscle repose and facilitate diagnostic procedures and surgical interventions [3, 4].

Dexmedetomidine, an active enantiomer of medetomidine, is the most potent alpha-2 adrenoceptor agonist with calming, analgesic, and muscle relaxing properties [5, 6]. Dexmedetomidine has beneficial pharmacological properties including its rapid distribution and half-life distribution, which encourages its use for equids. It allows rapid changes in the depth of sedation and rapid recovery after stopping its infusion [7].

Dexmedetomidine in donkeys, at a dose rate of 3–5 μg/kg, stimulated the sedation for 60 minutes with dose-based mechanical antinociception (40–55 minutes). Moreovere, dexmedetomidine at a 5 μg/kg dose may be therapeutically effective for mildly painful surgical procedures in standing sedation [8, 9].

Alpha-2 adrenoceptor agonists reduce gastrointestinal motility in the horse [10–12], which is clinically significant for horses suffering from gastrointestinal motility disorders. Furthermore, general anesthesia in horses causes gastrointestinal hypo-motility [13], which may predispose to conditions like ileus and caecal impaction [14, 15].

No previous studies have investigated the effect of dexmedetomidine on the gastrointestinal tracts of donkeys, unlike in rats there was previous research demonstrated the effect of dexmedetomidine on the rats’ gastric emptying and gastrointestinal transit [16]. Another study has assessed the effect of a low dose of dexmedetomidine on the gastrointestinal tracts of humans and revealed a decrease in gastric emptying rate [16, 17]. There is a paucity of data describing the gastrointestinal effects of different alagesic and sedative doses of dexmedetomidine in equines. The current study hypothesized that injection of dexmedetomidine at different doses would have an inhibitory effect on the gastrointestinal function in donkeys. Therefore, this research was designed to assess the impact of using the intravenous injection of dexmedetomidine at a dose of (3, 5, 7 μg/kg) on the intestinal peristaltic motility in healthy donkeys using transabdominal ultrasonography.

## Results

Clinical examination revealed that all the selected donkeys were clinically healthy throughout the experiment. There were no signs of infection at the needle puncture site, regional IV infusion site reaction, sudden onset hypersensitivity, and nervous system disorders throughout the observing time following IV isotonic saline solution or dexmedetomidine at different doses used. Each IV injection of isotonic saline solution had an analgesia score of 0 (0–0), manifested by a strong reaction to painful stimuli, a sedation score of 0 (0–0) is characterized by the donkeys being conscious, sensitive to noise, and environmental stimuli and an ataxia score of 0 (0–0) is characterized by the donkeys being able to walk without stumbling quickly.

Five miutes after the IV injection of dexmedetomidine at dose rates of 3, 5, and 7 μg/kg in the selected donkeys persuaded complete mutual perineal and tail analgesia, with a noted score 3 (3–3) until 30 minutes in all treatment groups. The level of analgesia was moderate in both 3 and 5 μg/kg groups with a noted score 2 (1–2) but the scores were higher in group 7 μg/kg with a noted score 3 (3–3) at 45 and 90 miuntes post dexmedetomidne injection (Table 1).

**Table 1:** Analgesia score median (range), post-intravenous injection of isotonic saline or Dexmedetomidine (3, 5, and 7 μg/kg) in Donkeys

Group	Time zero	5 minutes	15 minutes	30 minutes	45 minutes	60 minutes	90 minutes	120 minutes
**Normal saline**	0 (0–0) ^a^	0 (0–0) ^a^	0 (0–0) ^a^	0 (0–0) ^a^	0 (0–0) ^a^	0 (0–0) ^a^	0 (0–0) ^a^	0 (0–0) ^a^
**Dexmedetomidine (3** μg**/kg)**	0 (0–0) ^a^	3 (2–3) ^b^	3 (2–3) ^b^	3 (3–3) ^b^	2 (1–2) ^b^	1 (1–1) ^b^	1 (0–1) ^b^	0 (0–0) ^a^
**Dexmedetomidine (5** μg**/kg)**	0 (0–0) ^a^	3 (3–3) ^b^	3 (3–3) ^b^	3 (3–3) ^b^	3 (3–3) ^c^	2 (1–2) ^c^	1 (0–1) ^b^	0 (0–0) ^a^
**Dexmedetomidine (7** μg**/kg)**	0 (0–0) ^a^	3 (3–3) ^b^	3 (3–3) ^b^	3 (3–3) ^b^	3 (3–3) ^c^	3 (3–3) ^d^	2 (2–3) ^c^	1 (0–1) ^a^

^a,b,c,d^: Variables with different superscript letters in the same column are significantly different at *P* < 0.05

Mild sedation manifested by intermittent retort to external stimuli, lethargy, and minor drop of the head, eyelids, and lips in donkeys recorded post injection of 3 μg/kg dexmedetomidine, in which the sedation score was 1 (1–1). However, deep sedation, that was manifested by reducing animals’awareness, dropping head, lips, and eyelids, and decresing of response to external stimuli noted at 5 and 7 μg/kg of dexmedetomidine, in which the sedation score was 3 (2–3). Sedation was started at the 5 minutes and lasted until the 45 minutes at 3 μg/kg or 90 minutes at 5 and 7 μg/kg post dexmedetomidine administration (Table 2).

**Table 2:** Sedation score median (range), post-intravenous injection of isotonic saline or Dexmedetomidine (3, 5, and 7 μg/kg) in Donkeys

Group	Time zero	5 minutes	15 minutes	30 minutes	45 minutes	60 minutes	90 minutes	120 minutes
**Normal saline**	0 (0–0) ^a^	0 (0–0) ^a^	0 (0–0) ^a^	0 (0–0) ^a^	0 (0–0) ^a^	0 (0–0) ^a^	0 (0–0) ^a^	0 (0–0) ^a^
**Dexmedetomidine (3 μg/kg)**	0 (0–0) ^a^	2 (1–2) ^b^	2 (2–2) ^b^	2 (2–3) ^b^	1 (1–1) ^b^	1 (0–1) ^b^	0 (0–1) ^a^	0 (0–1) ^a^
**Dexmedetomidine (5 μg/kg)**	0 (0–0) ^a^	3 (2–3) ^c^	3 (3–3) ^c^	3 (3–3) ^c^	2 (2–2) ^c^	2 (1–2) ^c^	1 (1–2) ^c^	0 (0–1) ^a^
**Dexmedetomidine (7 μg/kg)**	0 (0–0) ^a^	3 (3–3) ^c^	3 (3–3) ^c^	3 (3–3) ^c^	3 (2–3) ^d^	2 (1–2) ^c^	1 (1–2) ^c^	0 (0–1) ^a^

^a,b,c,d^: Variables with different superscript letters in the same column are significantly different at *P* < 0.05

Moderate ataxia, and stumbling walking began at 5 minutes in all dexmedetomidine goups in which the ataxia score was 2 (2–2). Ataxia lasted up to 15 minutes after administration for 3 μg/kg, and up to 30 minutes after administration for 5 μg/kg, and up to 45 minutes after administration for 7 μg/kg (Table 3).

**Table 3:** Ataxia score median (range), post-intravenous injection of isotonic saline or Dexmedetomidine (3, 5, and 7 μg/kg) in Donkeys

Group	Time zero	5 minutes	15 minutes	30 minutes	45 minutes	60 minutes	90 minutes	120 minutes
**Normal saline**	0 (0–0) ^a^	0 (0–0) ^a^	0 (0–0) ^a^	0 (0–0) ^a^	0 (0–0) ^a^	0 (0–0) ^a^	0 (0–0) ^a^	0 (0–0) ^a^
**Dexmedetomidine (3 μg/kg)**	0 (0–0) ^a^	2 (2–2) ^b^	2 (2–2) ^b^	1 (1–1) ^b^	1 (1–1) ^b^	0 (0–0) ^a^	0 (0–0) ^a^	0 (0–0) ^a^
**Dexmedetomidine (5 μg/kg)**	0 (0–0) ^a^	2 (2–2) ^b^	2 (2–2) ^b^	2 (2–2) ^b^	1 (1–1) ^b^	1 (1–1) ^b^	0 (0–0) ^a^	0 (0–0) ^a^
**Dexmedetomidine (7 μg/kg)**	0 (0–0) ^a^	2 (2–2) ^b^	2 (2–2) ^b^	2 (2–2) ^b^	2 (2–2) ^b^	1 (1–2) ^b^	1 (1–1) ^b^	0 (0–0) ^a^

^a,b,c^: Variables with different superscript letters in the same column are significantly different at *P* < 0.05

Small and large intestinal contractions of donkeys showed no statistically significant differences between all groups at base time (zero-time; Table 4).

**Table 4:** The duodenal, jejunal, left colonic, right colonic, and cecal motility (contraction / 3 minutes) at zero-time pre- isotonic saline or Dexmedetomidine (3, 5, and 7 μg/kg) injection in donkeys

Group	Duodenal motility	Jejunal motility	Left colonic motility	Right colonic motility	Cecal motility
**Normal saline**	6.9 ± 1.3	6.2 ± 0.9	5.1 ± 1.2	6.9 ± 1.3	4.1 ± 1.3
**Dexmedetomidine (3 μg/kg)**	6.8 ± 1.2	6.2 ± 1.0	5.1 ± 1.0	6.8 ± 1.0	4.1 ± 1.0
**Dexmedetomidine (5 μg/kg)**	6.8 ± 1.2	6.2 ± 1.0	5.1 ± 1.0	6.8 ± 1.0	4.1 ± 1.0
**Dexmedetomidine (7 μg/kg)**	6.8 ± 1.2	6.2 ± 1.0	5.1 ± 1.0	6.8 ± 1.0	4.1 ± 1.0^a^

The results of the two-way repeated measures ANOVA demonstrated a statistically significant (*P* < 0.01) effect of time and both treatments for the contractility of each of the duodenum, jejunum, left colon, right colon, and cecum, implying that the impact of treatment on gastric motility varied among different time points.

**Fig. 1:**
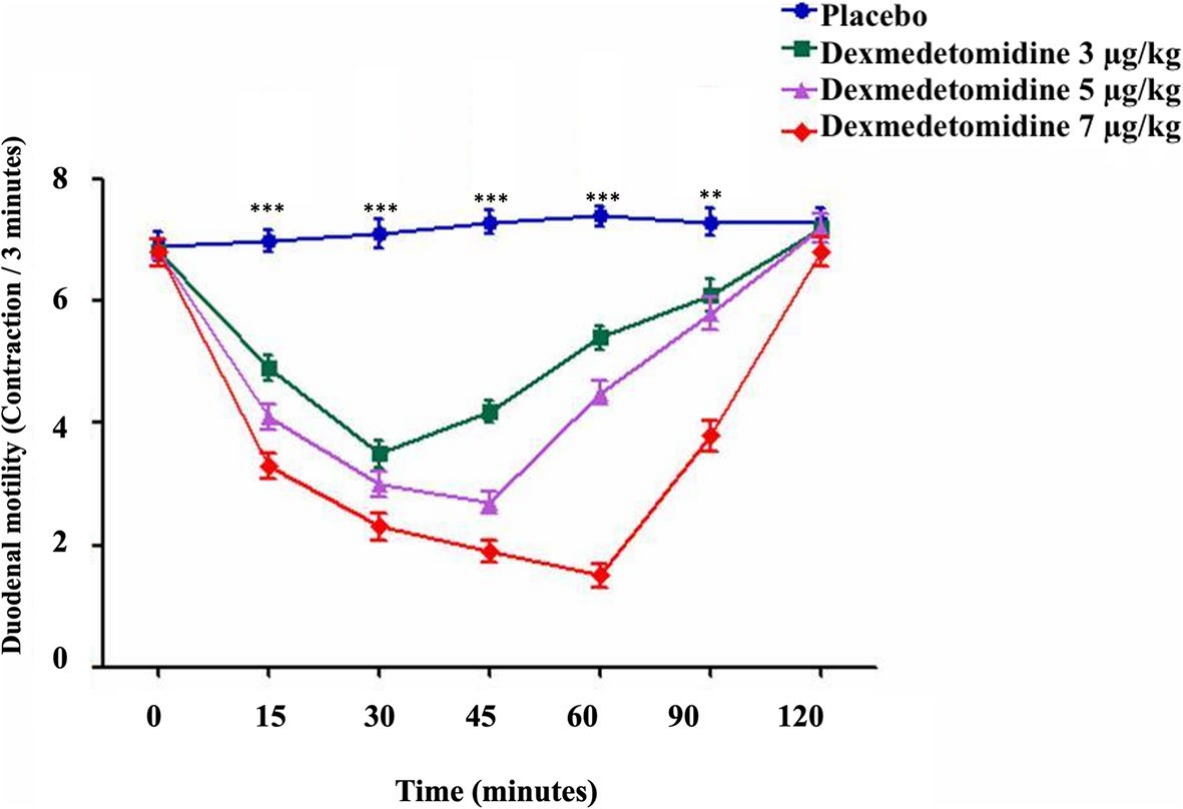
The duodenum motility in healthy donkeys (*Equus asinus*) after IV administration of isotonic saline or dexmedetomidine at 3, 5, and 7 μg/kg. Each point represents the number of contractions (contraction / 3 minutes) expressed as mean ± standard deviation (SD) at different time points zero, 15, 30, 45, 60, 90, and 120 minutes post-administration. *: Mean ± SD with a superscript asterisk at the same time point are significantly different at *P* < 0.01

**Fig. 2:**
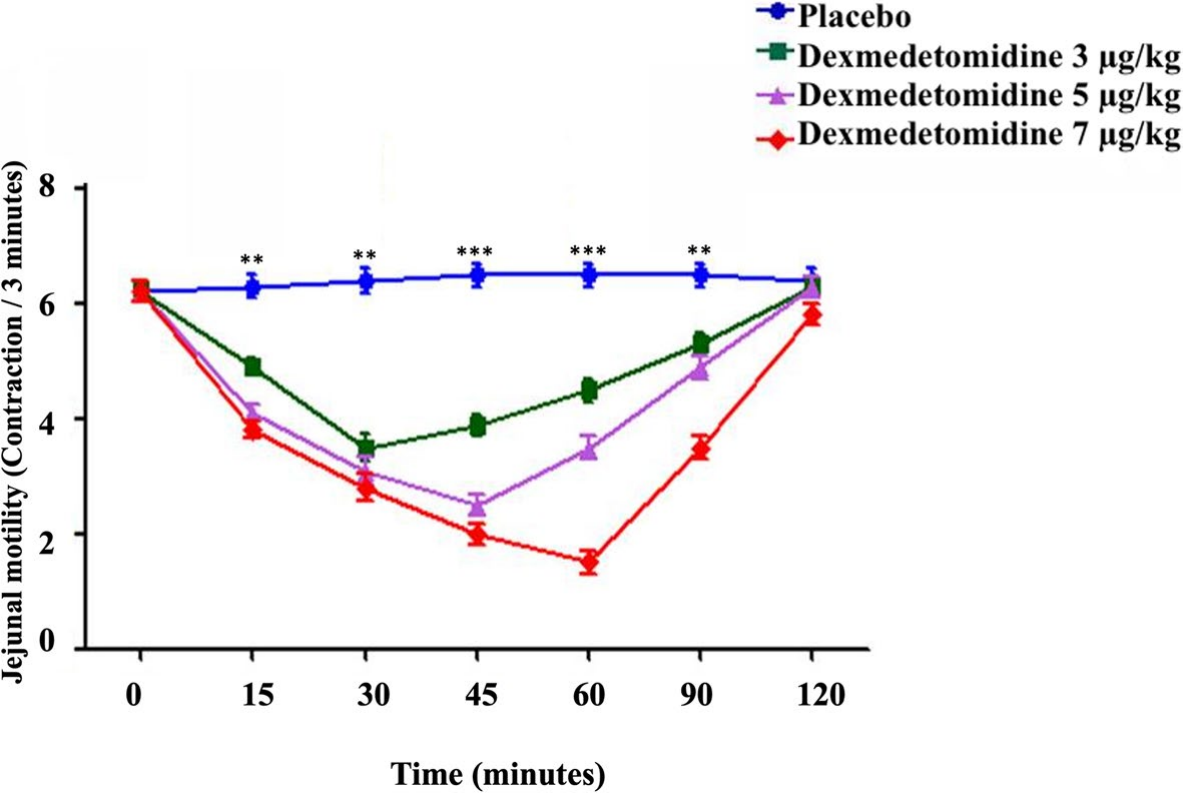
The jejunal motility in healthy donkeys (*Equus asinus*) after IV administration of isotonic saline or dexmedetomidine at 3, 5, and 7 μg/kg. Each point represents the number of contractions (contraction / 3 minutes) expressed as mean ± standard deviation (SD) at different time points zero, 15, 30, 45, 60, 90, and 120 minutes post-administration. *: Mean ± SD with a superscript asterisk at the same time point are significantly different at *P* < 0.01

**Fig. 3:**
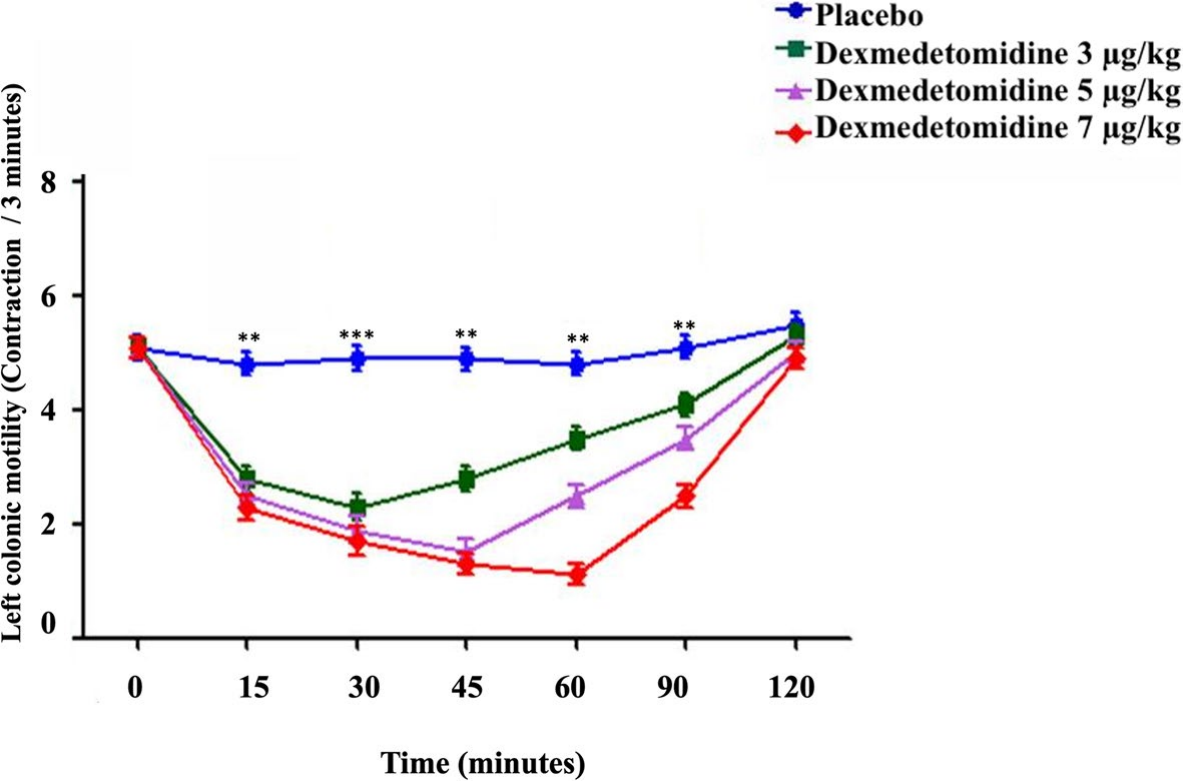
The left colonic motility in healthy donkeys (*Equus asinus*) after IV administration of isotonic saline or dexmedetomidine at 3, 5, and 7 μg/kg. Each point represents the number of contractions (contraction / 3 minutes) expressed as mean ± standard deviation (SD) at different time points zero, 15, 30, 45, 60, 90, and 120 minutes post-administration. *: Mean ± SD with a superscript asterisk at the same time point are significantly different at *P* < 0.01

**Fig. 4:**
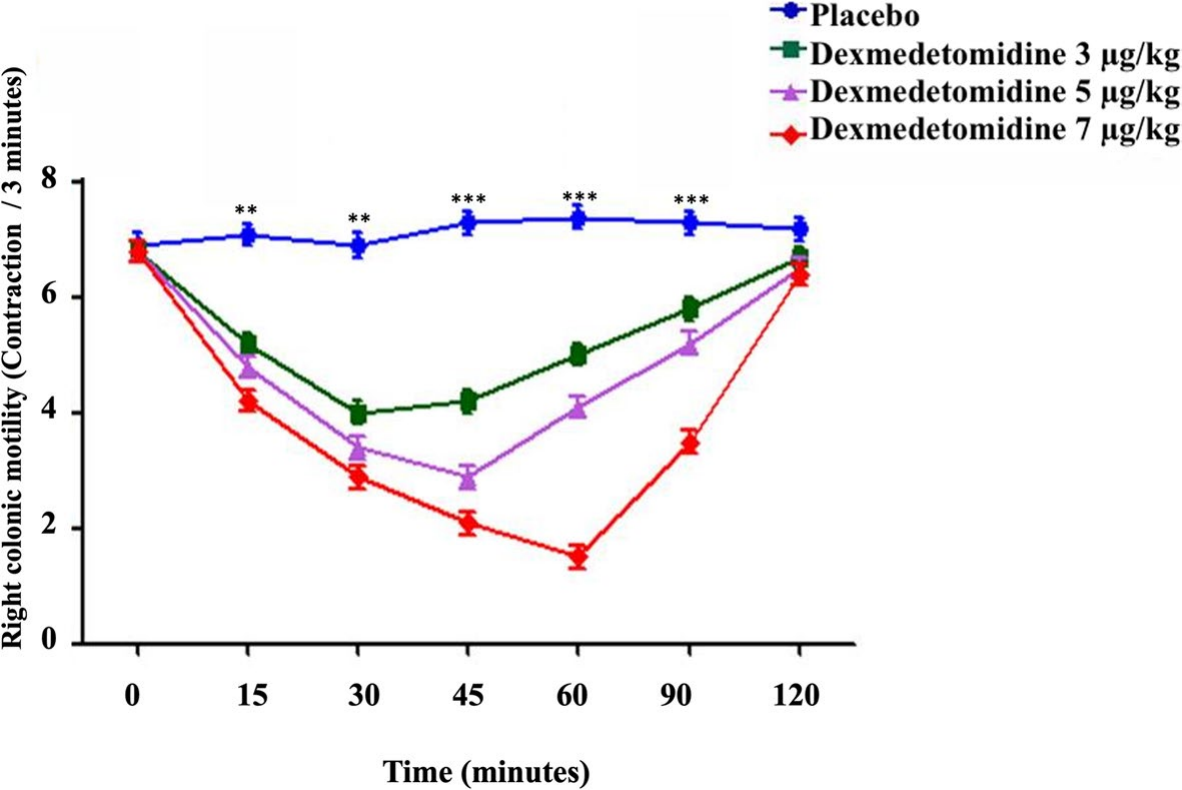
The right colonic motility in healthy donkeys (*Equus asinus*) after IV administration of isotonic saline or dexmedetomidine at 3, 5, and 7 μg/kg. Each point represents the number of contractions (contraction / 3 minutes) expressed as mean ± standard deviation (SD) at t different time points zero, 15, 30, 45, 60, 90, and 120-minutes post-administration. *: Mean ± SD with a superscript asterisk at the same time point are significantly different at *P* < 0.01

**Fig. 5:**
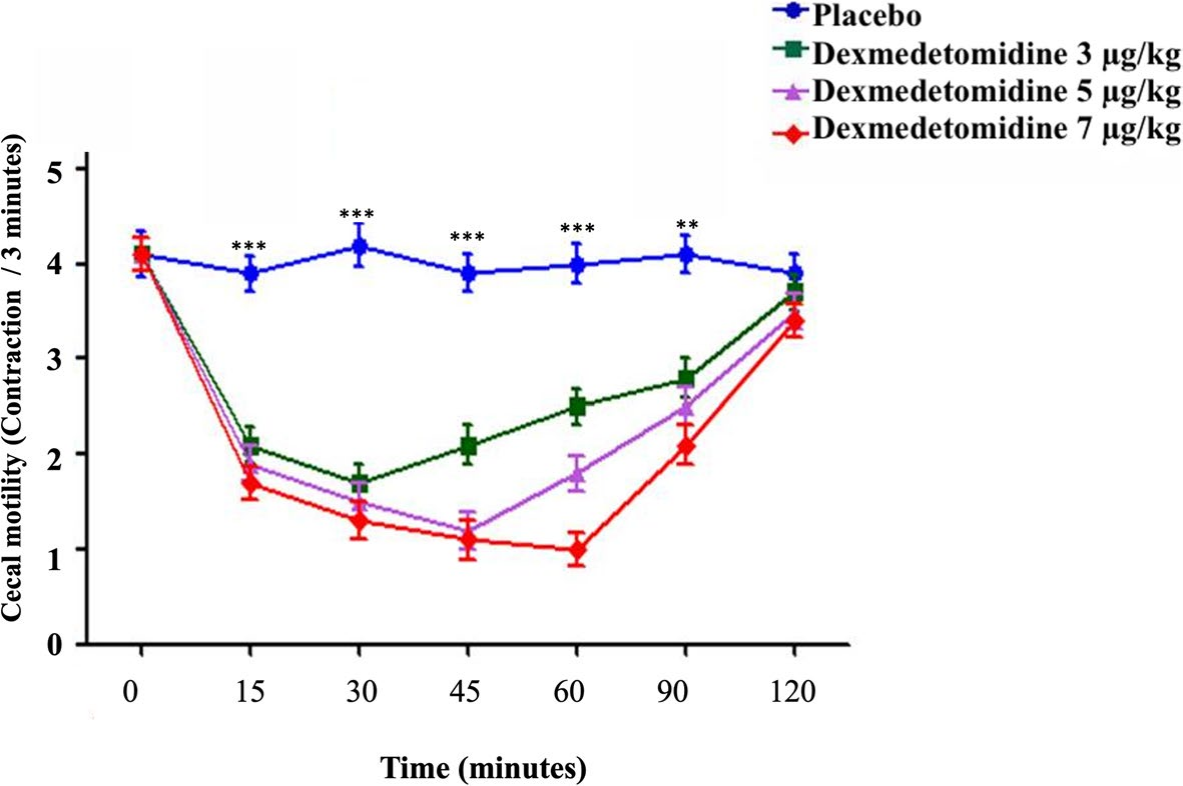
The cecum motility in healthy donkeys (*Equus asinus*) IV administration of isotonic saline or dexmedetomidine at 3, 5, and 7 μg/kg. Each point represents the number of contractions (contraction / 3 minutes) expressed as mean ± standard deviation (SD) at t different time points zero, 15, 30, 45, 60, 90, and 120 minutes post-administration. *: Mean ± SD with a superscript asterisk at the same time point are significantly different at *P* < 0.01

The simple main effect analysis revealed that the IV dexmedetomidine at 3, 5, and 7 μg/kg doses significantly altered bowel contractility compared to administration of isotonic saline (*P* ≤ 0.01). After IV injection of normal saline in the donkey under experiments, the contractility of each of the examined portions of the small and large intestine did not significantly fluctuate during the 2 h driving period and stayed within the typical levels until 120 minutes post-administration. Whereas the contractility of each of the examined portions of the small and large intestine was changed post IV injection of dexmedetomidine in the chosen donkeys (Figs. 1, 2, 3, 4 and 5).

At 15, 30, 45, and 60 minutes after injection, intravenous dexmedetomidine at 3 μg/kg caused a significant decrease in both duodenal (*P* ≤ 0.003) and jejunal motility compared to placebo (*P* ≤ 0.005). At 30 minutes after administration, the minimum contractions (contraction / 3 minutes) of both duodenum and jejunum were 3.5 ± 1.2 and 3.5 ± 1.3, respectively (Figs. 1 and 2). Nevertheless, dexmedetomidine at 5 and 7 μg/kg doses caused a significant reduction in both duodenal (*P* ≤ 0.003) and jejunal (*P* ≤ 0.005) motility frequencies compared to placebo at 15, 30, 45, 60, and 90 minutes post-injection. The minimum contractions (contraction / 3 minutes) of both duodenum and jejunum after IV dexmedetomidine (5 μg/kg) were 2.7 ± 1.0 and 2.5 ± 1.0, respectively, which were noted at 45 minutes post-administration (Figs. 1, 2). The minimum contractions (contraction / 3 minutes) of both duodenum and jejunum after IV dexmedetomidine at 7 μg/kg were 1.5 ± 1.1 and 1.5 ± 1.1, respectively, which were noted at 60 minutes post-administration (Figs. 1 and 2).

The left colon showed significantly decreased motility at 15, 30, and 45 minutes post IV injection of 3 μg/kg of dexmedetomidine compared to placebo (*P* ≤ 0.009). The minimum contractions (contraction / 3 minutes) of the left colon motility after IV dexmedetomidine at 3, 5 and 7 μg/kg were 2.3 ± 1.3, 1.5 ± 1.2, and 1.1 ± 1.0 respectively, which were recorded at 30, 45, and 60 minutes post-injection (Fig. 3).

At 15, 30, 45, 60, and 90 minutes after injection, IV dexmedetomidine at 3, 5, and 7 μg/kg caused a significant decrease in right colon motility compared to placebo (*P* ≤ 0.004). The minimum contractions (contraction / 3 minutes) of the right colon motility after IV dexmedetomidine at 3, 5 and 7 μg/kg were 4.0 ± 1.1, 2.9 ± 1.1, and 1.5 ± 1.0, respectively, at 30, 45, and 60 minutes post-administration (Fig. 4).

Similarly, dexmedetomidine of 3 μg/kg caused a significant decline in the motility of cecum compared to placebo at 15, 30, 45, and 60 minutes post-injection (*P* ≤ 0.003). However, dexmedetomidine of 5 and 7 μg/kg caused a significant decline in the cecum motility compared to placebo at 15, 30, 45, 60, and 90 minutes post-injection. The minimum contractions (contraction / 3 minutes) of the motility of cecum after intravenous dexmedetomidine at a dose ratio of 3, 5, and 7 μg/kg were 1.7 ± 1.1, 1.2 ± 1.1, and 1.0 ± 1.0, respectively which were recorded at 30, 45, and 60 minutes post-administration (Fig. 5).

## Discussion

The results of the current study showed that intravenous injection of dexmedetomidine at 3, 5, and 7 μg/kg significantly inhibited of perstalitic movement of different intestinal segments. Dexmedetomidine is an α-2 adrenoreceptor agonist that is gaining interest as a part of the balanced anesthetic protocol in equine anesthesia. It provides deep sedation and has a minimum alveolar concentration sparing effect [18]. It may result in a higher quality of recovery than the other balanced protocols used in horses [19, 20]. Dexmedetomidine focuses on researchers’ attention to find out its adverse effects on various parts of the body, including the gastrointestinal tract. Therefore, this study is the first to investigate the effects of dexmedetomidine on the motility of both the small intestine (duodenum and jejunum) and the large intestine (left colon, right colon, and body of cecum) in donkeys (*Equus asinus*) using transabdominal ultrasonography.

The frequency of duodenal, jujenum, left colon, right colon and cecal contractions in donkeys are closely similar reported in horses [21, 22] and in the perivous study in [23].

In this current study, the analgesic effect of dexmedetomidine was observed at the 5 minutes and after IV administration and lasted up to the 30 minutes post-administration for 3 μg/kg dose, 45 minutes for 5 μg/kg, and 60 minutes for 7 μg/kg dose, consistent with the findings of previous studies [8, 9]. The dose of dexmedetomidine used in this investigation was determined based on prior equine studies [8, 24]. The sedative effect of dexmedetomidine was observed 5 minutes after its IV administration and lasted 60 minutes post-administration for 3 μg/kg dose and 90 minutes for both 5 and 7 μg/kg. These findings are comparable to those previously reported in donkeys, where increasing dexmedetomidine dosages from 4 to – 5 μg/ kg increased the sedation time from 30 to 60 minutes [8]. Dexmedetomidine also has a dose-dependent sedative effect that does not exceed a certain level [25]. Therefore, dexmedetomidine has a beneficial pharmacological profile, including rapid redistribution and a short half-life [18, 26]. There were significant differences between treatments for the analgesia and sedation scores. For the ataxia scores, there were no significant differences between treatments. This finding was confirmed by [27], which of demonstrated that a higher dose of epidural xylazine in equines has not been proven to induce ataxia. More research is needed to determine whether increasing dexmedetomidine doses causes substantial changes in ataxia scores.

The anatomical location and the ultrasonographic presence of the visualized sections of both the small and large intestine using abdominal ultrasonography agreed with those previously described [28, 29]. Before IV injection of normal isotonic saline or different selected doses of dexmedetomidine in the donkeys under study, the regularity of contractility of both small (duodenum and jejunum) and large (left colon, right colon, and cecum) intestines were within normal ranges, which are directly comparable those reported by [23, 28, 30]. The IV injection of isotonic saline solution in the donkeys did not influence the contractility of the visualized sections of the small and large intestine during 120 minutes motoring period and stayed inside the ordinary varies till 120 minutes post-injection as formerly described in humans [31]. The effect of different doses of dexmedetomidine on gastrointestinal motility was consistent across all donkeys at 90 minutes. Clonidine and dexmedetomidine are alpha 2 adrenoceptor agonists that induce sedation, reduce anesthetic and analgesic doses, and improve peri-operative hemodynamic balance [32]. Dexmedetomidine, unlike donkeys, inhibits gastric, small bowel, and colonic motility in animal and human studies [32, 33].

In the previous studies conducted on humans and animals, dexmedetomidine inhibited all gastrointestinal tract motor function segments. Its antiperistatical effects are due to the inhibition of excitatory cholinergic pathways in the enteric nervous system via 2-adrenoceptors or activated inhibitory neural pathways [21, 34–36]. Dexmedetomidine is a promising agent for palliative sedation due to its unique mechanism of action, which causes dose-dependent sedation without a significant risk of respiratory depression [4, 35]. In horses, the decreased gastrointestinal motility was an anticipated finding following administration of dexmedetomidine, which is one of the negative effects of α2-adrenoceptor agonists on equine gastrointestinal motility, which has been extensively described in the literature [37, 38]. Furthermore, detomidine and medetomidine decreased gastrointestinal motility in horses for 120 and 90 minutes [38], respectively. While in the current study, the inhibition effect of dexmedetomidine on the donkey’s gastrointestinal motility lasted only 60 minutes [39]. demonstrates that donkeys appear to metabolize many anesthetic and sedative drugs differently than horses.

Based on these findings, intravenous injection of dexmedetomidine in the studied donkeys resulted in a significantly decline in the motility of the duodenum, jejunum, left colon, right colon, and cecum when compared to placebo. The greatest inhibitory effect was done at dose 7 μg/kg and take a long obvesration period than other treatments. The noticible point appears to be cecal motility was the most affected than other intestinal parts motility in healthy donkeys. In donkey, the effect of IV dexmedetomidine began at the 5 minutes and lasted up to 30–90 minutes based on the dose given. The results of pharmacokinetics studies revealed that dexmedetomidine concentrations decreased rapidly with an elimination half-life ranging between 7.19 and 8.87 minutes, and the last detection time varied between 30 and 60 minutes. The plasma concentrations of dexmedetomidine peaked 1–4 minutes post-administration [35].

The current study’s limitations necessitate further research to investigate the pharmacokinetics of dexmedetomidine in donkeys. In addition, since our study’s grading system is subjective, it may not accurately assess sedative, analgesic, and ataxic effects. The same person who measured analgesia, sedation, and ataxia was blinded to the medication administered to overcome this limitation. As the current study was performed on healthy donkeys depending on the investigational design, the results obtained may not reflect the actual characteristics of diseased donkeys with disturbed gastrointestinal tract motor function. Consequently, more research is needed to determine the influence of this drug in donkeys with impaired gastrointestinal tract motor function.

## Conclusion

The current study revealed that IV administration of dexmedetomidine at different recommended sedative doses caused a potent inhibitory effects on the small and large intestinal perstatlic movenet in healty donkeys “*Equus asinus*”. Consequently, it may be beneficial to raise awareness of this potential effect, particularly when used in equines with disturbed gastrointestinal tract motility.

## Methods

### Study sample

This experimental study included 30 healthy donkeys (*Equus asinus*) (15 males and 15 females) aged between 5 to 9 years old and weighing between 100 to 220 kg. The inclusion criteria for the selected donkeys were (1) clinically healthy, (2) free from any gastrointestinal disorders, (3) free from any evidence of other systemic diseases, and (4) easily manageable without any sedation. These donkeys were purchased from Dakahlia province (Egypt). They were in the stall’s interior of the animal barn for 2 weeks prior to the study. On arrival, the donkeys were immunized and dewormed with ivermectin glue (Bimectin®, Bimeda Animal Health Ltd., Ireland) at a dosage rate of 0.2 mg/kg. The feeding regimen for the selected donkeys was a uniformly balanced share comprising sliced wheat straw ad libitum, grain (1.5 kg), and crushed corn (1.5 kg), supplemented with all the necessary trace elements and minerals. The diet was offered twice daily at fixed times; 7.00 am and 7.00 pm to reduce the effect of the type of the diet on the contractility of the gastrointestinal tract.

Furthermore, animals had free access to tap water. The Animal Welfare and Ethics Committee of the Faculty of Veterinary Medicine, Code No. R/63 validated all animal care and testing procedures following the Guidelines for Animal Use and Care published by the Faculty of Veterinary Medicine, Mansoura University, Egypt.

### Study design

Each donkey was randomly assigned to one of four trials, with a two-week washout period, which began 1 h after feeding. The first group (placebo) received an IV of 20 mL of normal isotonic saline. The second, third, and fourth groups (treatment groups) were treated with dexmedetomidine hydrochlorid (Precedex®, Lakeforest, USA) at the dosage of 3, 5, and 7 μg/ kg IV. respectively. For sedation doses, the medication was diluted with sodium chloride to a total volume of 20 mL after preparing the required dose of dexmedetomidine for each donkey. One-third of the dose was administered as an IV bolus, with the remaining two-thirds being injected slowly over 2 minutes.

In the experiment, donkeys, analgesia, sedation, and ataxia were measured using 0 to 3 scoring system, as previously stated [40]. Analgesia was proven with deep muscle pinpricking with a 2.5-cm-long hypodermic needle. The needle was repeatedly inserted into the underlying tissues via the skin of the neck, shoulder region, coronary band, paralumbar fossa, and hip area. As progressive pain signals, repetitive head, neck, trunk, limb, and tail movements to avoid the needle and attempts to kick and rotate the head to the painful site were observed. The needle was placed in slightly different bilateral positions for each test, ranging from caudal to cranial. The period from drug administration to sensation impairment was defined as the time of effect onset. The time between the disappearance and recurrence of pinprick stimuli was defined as the antinociceptive duration. The degree of analgesia was graded from 0 to 3: 0 = no analgesia (strong reaction to harmful stimuli, like kicking); 1 = mild analgesia (mild reaction, such as shifting the heads towards the stimulus spot); 2 = moderate analgesia (minimal and recurring reaction); and 3 = complete analgesia (no response to noxious stimulation). The degree of sedation was rated on a scale of 0 to 3: 0 = no sedation (donkeys maintained their original attitude and were sensitive to noise and stimulus); 1 = mild sedation (reduced attention with slight responses to external stimulation, irregular stumbling, and the ability to resume walking); 2 = moderate sedation (somnolence, dullness, and occasional response to external stimuli; slight sunken of the head, lips, and upper eyelids; and marked stumbling and walking); and 3 = deep sedation (recumbence or collapsing while walking; obvious lethargy, head droop, and failure to respond to environmental cues). The degree of ataxia was graded from 0 to 3 as follows; 0 = normal; 1 = mild (slight stumbling but quickly able to walk afterward); 2 = moderate (observable stumble and apparent ataxic walk); 3 = extreme (recumbency or landing while walking). The same person who measured analgesia, sedation, and ataxia was blinded to the medication administrated. The degree of analgesia, sedation, and ataxia was measured before injection (time zero) and at 5, 15, 30, 45, 60, 90, and 120 minutes after injection. Since the solid phase of gastric discharging begins within 30 minutes of eating, each trial in this study began 1 hour after the donkeys had finished eating.

The motility of each of the duodenum, jejunum, left colon, right colon, and cecum was measured over 3 minutes via trans-abdominal ultrasonography before administration (time zero) and at 15, 30, 45, 60, 90, and 120 minutes after injection of the drug. The measuring unit is (contraction / 3 minutes). The donkeys were not given food or water during the ultrasound scanning.

### Transabdominal ultrasonography

The abdominal region expanding from the seventh intercostal space backward up to the lumbar fossa was bilaterally clipped and prepared for the ultrasonographical examination. The coupling gel was applied to those areas, and a linear transducer (2.5–5 MHz) (iVis 60 Expert Vet®, Chison Medical Imaging Co. Ltd., China) was selected. The scan depth was initially set to maximum penetration and then adjusted to different depths based on the scanned individual structure to obtain the best definition of structures and maximize image quality. The left colon and jejunum were scanned from the left abdominal wall, and the duodenum, right colon, and cecum were examined from the right abdomen. As previously stated [28, 29], the physiological position and structure of the ultrasound image were used to identify the specific parts of the intestine in each donkey. All ultrasound procedures to quantitatively assess the motility of the selected parts of the intestine were initiated 1 h after finishing eating (the first meal) and were done by the same person to prevent any variations and reviewed by two experts.

### Data analysis

Data were analyzed using the SPSS software for Windows, version 21.0; IBM Inc., Chicago, IL). The normally distributed were analyzed based on the Kolmogorov–Smirnov test output. The non-parametric Kruskal–Wallis test with post hoc Dunn’s multiple comparison test was used at various time points to evaluate statistical differences between evaluated parameters (analgesia, sedation, and ataxia) treatments. For parametric data of the intestinal contractility frequencies, two-way repeated measures ANOVA was used to evaluate the impact of time, treatment, and interaction between time and treatment. Wilks’ lambda test was utilized to evaluate within-group and time x treatment binding evidence. Meanwhile, Wilks’ lambda test revealed a statistically significant difference between groups. The One-Way ANOVA test was used to determine which group was statistically different at each time point. The data were presented in run charts of the intestinal cramp during the observation period in both experiments. The level of statstcal significance was determined at *P* < 0.05 in all statistical analyses.

## Data Availability

All data generated or analyzed during this study are included in this article.
